# Charging and discharging at the nanoscale: Fermi level equilibration of metallic nanoparticles

**DOI:** 10.1039/c5sc00461f

**Published:** 2015-03-23

**Authors:** Micheál D. Scanlon, Pekka Peljo, Manuel A. Méndez, Evgeny Smirnov, Hubert H. Girault

**Affiliations:** a Laboratoire d'Electrochimie Physique et Analytique , Ecole Polytechnique Fédérale de Lausanne , Station 6 , CH-1015 Lausanne , Switzerland . Email: huber.girault@epfl.ch; b Department of Chemistry , Tyndall National Institute , University College Cork , Cork , Ireland

## Abstract

The redox properties of metallic nanoparticles are discussed, in particular the relationships between excess charge, size and the Fermi level of the electrons. The redox potentials are derived using simple electrostatic models to provide a straightforward understanding of the basic phenomena. The different techniques used to measure the variation of Fermi level are presented. Finally, redox aspects of processes such as toxicity, electrochromicity and surface plasmon spectroscopy are discussed.

## Abstract 2

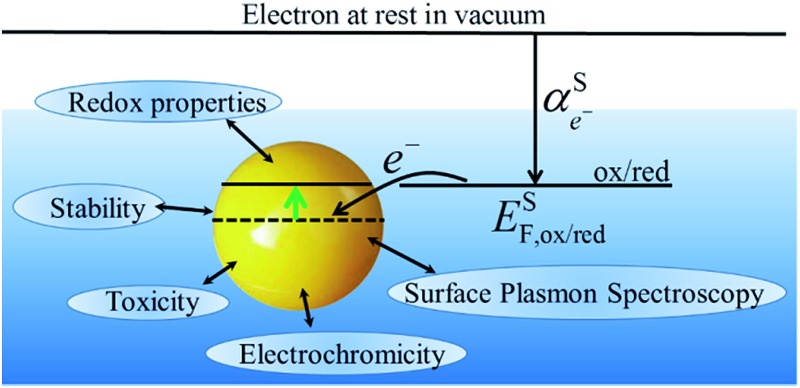
Surrounding environment, excess charge and size affect the Fermi level of the electrons in nanoparticles, having a significant influence on their properties.

## Introduction

From an electrochemical viewpoint, metallic nanoparticles (NPs) can be regarded as multivalent redox species with a wide range of redox states that may be charged or discharged by interaction with their environment.

The redox properties of metallic NPs, especially those of gold (Au) and silver (Ag), have been extensively studied and may be divided into three distinct voltammetric regimes based on their sizes: (i) bulk continuum, (ii) quantized charging and (iii) molecule-like voltammetry.^1,2^ For the largest metallic NPs, with core sizes typically in the range 2 to 100 nm, the redox potentials of each charge state are so close together that they form a continuum. As the core size of the NPs decreases to less than 2 nm, the NPs are renamed “nanoclusters (NCs)” and a threshold is reached where the redox potentials of the different charge states are separated enough or “quantised” such that they may be measured distinctly. Such measurements have been achieved with metallic NCs of gold, copper and various alloys coated with an organic monolayer of alkanethiols and are commonly referred to as monolayer-protected clusters (MPCs).^3^

In this perspective, we will focus on the shifts of Fermi level and its influence on the chemical and electrochemical properties of NPs. For further information on the synthesis of sub-nanometer sized metal NCs and their interesting catalytic, fluorescent and chiral properties, the reader is referred to recent reviews.^4,5^ Additionally, we will limit the scope to metallic NPs, and not semiconductor nanocrystals or quantum dots, as reviewed and discussed in detail elsewhere.^6–8^

## The Fermi level of an electron in solution

The electrochemical potential of an electron in an aqueous solution 
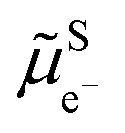
 is a concept associated with the presence of a redox couple (ox/red) in solution (S) and the following virtual redox reaction between an electron and that redox couple: ox^S^ + e^–S^ ⇄ red^S^. At equilibrium, we can define the electrochemical potential for the virtual electron in solution as the difference between the electrochemical potentials of the reduced and oxidised species, respectively. It represents the work to bring an electron at rest in vacuum to the solution containing the redox couple.^9^
1
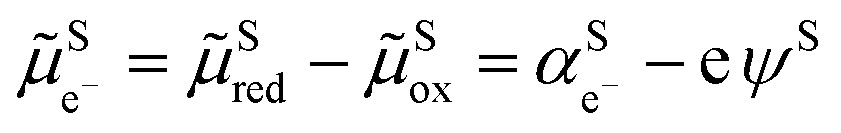
where *α* is the real chemical potential and *ψ* ^S^ is the outer potential associated with the presence of excess charge on the solution. By analogy with the Nernst equation, we can define the standard redox potential on the absolute vacuum scale (AVS) by considering the virtual reduction reaction between an electron at rest in vacuum and the oxidised species in solution: ox^S^ + e^–V^ ⇄ red^S^ to have

2 *e*[*E*_ox/red_]SAVS = –Δ*G*_red_ = *μ̃*Sox – *μ̃*Sred

as by definition the electron at rest in vacuum is the origin of the AVS scale 
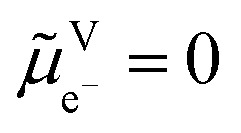
.

By comparing eqn (1) and (2), we can define the electrochemical potential of the electron in solution and consequently define a Fermi level for the electron in solution as shown in Scheme 1.3
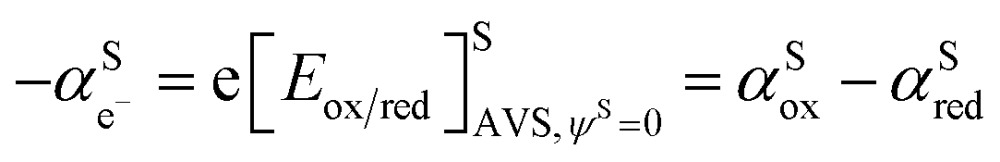
Eqn (3) states that the Fermi level of an electron in solution depends on the real potential of ox and red. In the case of a system with multiple redox couples, eqn (3) has to be fulfilled for all the redox active species in equilibrium, and typically one redox species in excess will dominate the Fermi level of the solution.

**Scheme 1:**
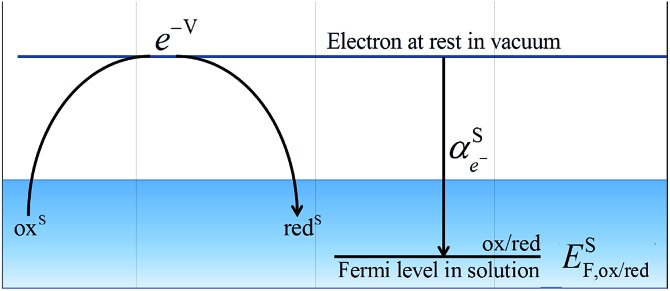
Redox reaction for the definition of the absolute redox potential considering electrons at rest in vacuum.

## The ionization energy of a metallic NP in vacuum

The work function *Φ* is the work to remove an electron from a neutral and large piece of metal, whereas the ionization energy is a term usually associated with atoms and molecules but also charged NPs for the extraction of an electron: NPV*ze* → NPV(*z*+1)*e* + e^–V^. The ionization energy IE in vacuum of a spherical metallic NP of charge *ze* and radius *r* can be expressed using elementary electrostatics^10–12^
4
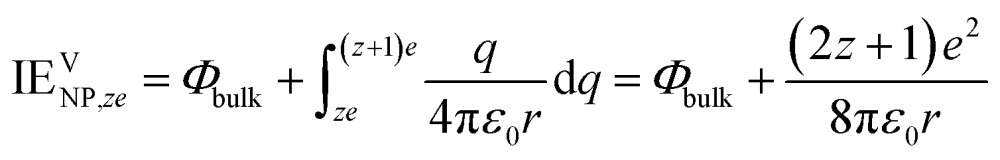
IEVNP,*ze* contains a bulk term for the work function, and a charging term. More generally, the ionisation energy of a neutral NP has been proposed to read5
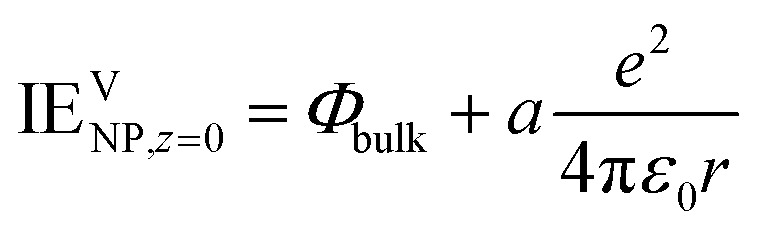
where the coefficient *a* can be considered to be equal to 1/2 as in eqn (4) or 3/8 according to the electrostatic model used.^13^ A recent review by Svanqvist and Hansen^14^ compared both experimental and computational values of work functions of small clusters, and concluded that metals tended to have a coefficient *a* of *ca.* 0.3. This variation of the coefficient *a* is due to quantum effects.^14^

**Scheme 2:**
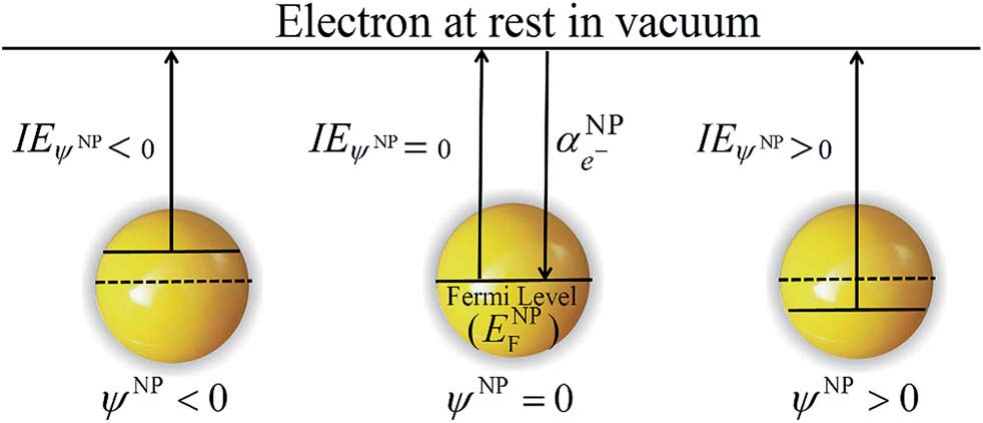
Representation of the apparent ionization energy for the extraction of an electron from a metallic NP in vacuum when the surface of the metallic NP is neutral (*ψ* ^NP^ = 0), negatively (*ψ* ^NP^ < 0) and positively (*ψ* ^NP^ > 0) charged.

 Eqn (4) shows that as a metallic NP becomes more negatively charged (*z* < 0), its ionization energy in vacuum decreases as the energy required to extract an electron decreases. Inversely, as a metallic NP becomes more positively charged (*z* > 0), its ionization energy increases as illustrated in Scheme 2.

 Eqn (4) also demonstrates that the ionization energy of a neutral metallic NP in vacuum is higher than the corresponding work function of the bulk metal. Hence, electrons in neutral metallic NPs are at a lower Fermi level than in the bulk metal. In vacuum, the ionisation energy of a neutral Au NP lies somewhere between that of a gold atom, 9.2 eV,^15^ and the work function of bulk gold metal, approximately 5.3 eV.^9^

It is important to note that the charge on the NP could be electronic or electrostatic due to the presence of adsorbed ionic species or ligands. The difference between these ionization energies under neutral and charged conditions is directly related to the excess charge on the metallic NP and, therefore, on the outer potential.

6 IE_*ψ*^NP^≠0_ – IE_*ψ*^NP^=0_ = *eψ*^NP^

For spherical metallic NPs, the outer potential is directly related to the excess charge *ze* by the capacitance and is given by:7
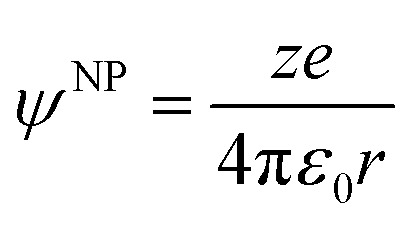



If we consider a nanoparticle on a support, the capacitance will depend on the geometry of the support.

## The Fermi level and redox potential of a metallic NP in solution

**Fig. 1:**
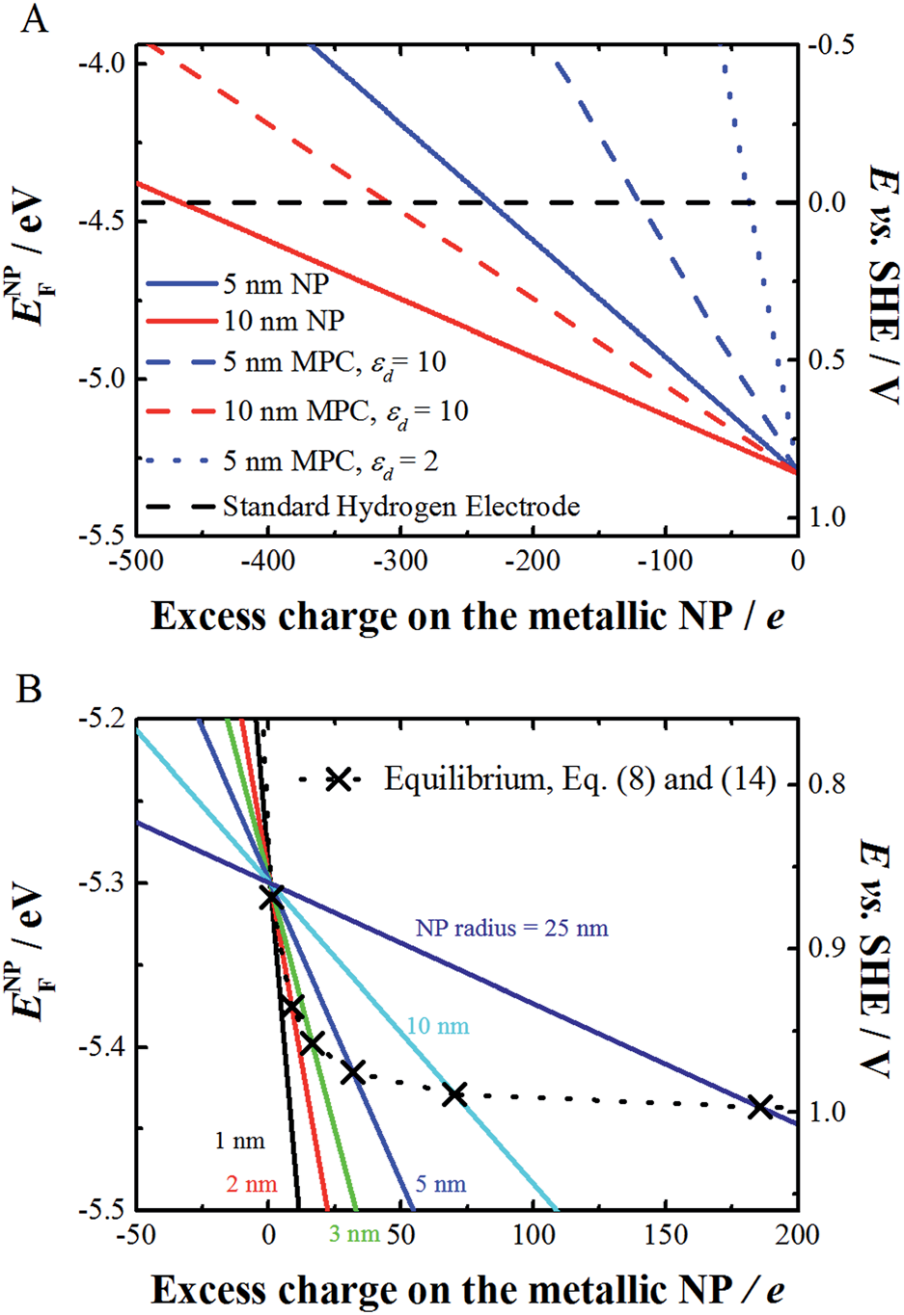
(A) Variation of the Fermi level of 5 and 10 nm radius bare (eqn (8), solid line, *d* = *ε*_d_ = 0) and monolayer protected (eqn (8), dashed or dotted line, *d* = 0.8 nm and *ε*_d_ = 10 or 2) spherical Au NPs as a function of increasing charge on the metallic NP in solution (in this case water). (B) Variation of the Fermi level of bare metal NPs of different radius and the corresponding equilibrium potential between the Au NP and AuCl_4_^–^ ions, as calculated using eqn (8) and (14) when the activities of Au NPs and AuCl_4_^–^ are taken as unity.

The redox potentials of a metallic NP can be evaluated with thermodynamic cycles, as previously shown by Su and Girault.^11^ For the reduction of a metallic NP in solution:8
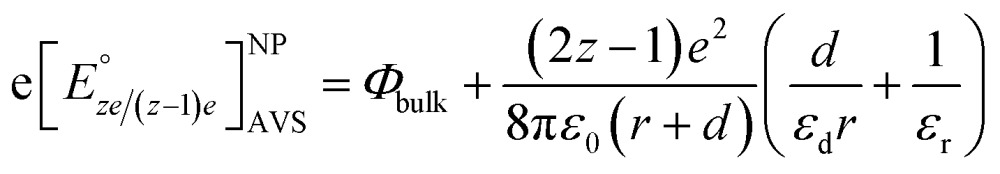
where *d* and *ε*_d_ are the thickness and relative permittivity of an adsorbed layer. Eqn (8) shows that the absolute standard redox potential of a spherical, metallic and chemically inert NP depends on the work function of the bulk metal but also on a term that takes into account the size and charge of the metallic NP and the dielectrics of the solvent and an adsorbed molecular layer (if present). Fig. 1A compares the redox potentials of bare and layer-coated NPs of radii 5 and 10 nm in solution, with *d* = 0.8 nm, and *ε*_d_ = 10 or 2 and taking *Φ*_Au_ as 5.3 eV. It is worth noting that the charging of these metallic NPs is not quantized but strongly depends on the presence of an adsorbed layer. The capacitance of these larger metallic NPs (>5 nm) is rather small, and hence the change of charge by variation of one electron results in very a small variation of the Fermi level of the NP, *E*NPF. The slopes in Fig. 1A represent the reciprocal of the capacitance.

As gold is a noble metal, *E*NPF for an uncharged particle is well below that of the Fermi level for the H^+^/H_2_ redox couple (taken here equal to –4.44 eV). Indeed, Fig. 1 shows that *E*NPF remains more negative than this value unless the charge on the NP becomes largely negative. Approximately 500 negative charges are needed on a 10 nm Au NP, corresponding to a charge density of 64 mC m^–2^ (for comparison silica has a charge density of 10 mC m^–2^ at neutral pH) to reach 0 V *vs.* the Standard Hydrogen Electrode (SHE). For comparison, nanorods have been charged to a charge density of 2100 mC m^–2^.^16^

Metallic NPs in solution are often synthetized with anions adsorbed on the NPs (*e.g.* citrate anions in the Turkevich synthesis of gold NPs). It is important to note that the adsorbed ionic charges contribute to the position of *E*NPF, and the charge *ze* in eqn (8) includes both the excess number of electrons and the adsorbed ionic charges.

To keep here a simple electrostatic model considering the solvent as a dielectric continuum, the capacitance of an MPC is given by:^17^
9
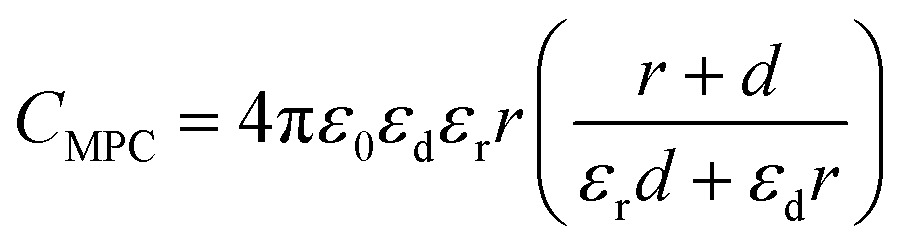
this equation is self-consistent with eqn (8) when calculating the separation of the redox potentials upon charging. This model can be extended to take into account the effects of the diffuse electrical double layer surrounding the MPC (by both linearized and non-linear Poisson–Boltzmann (P–B) models),^18–20^ and also the solvent and ion penetration into the surrounding monolayer.^21,22^ In fact, the capacitance of an MPC can be considered as two capacitors in series, one for the monolayer of thickness *d* and one for the bulk solution.10
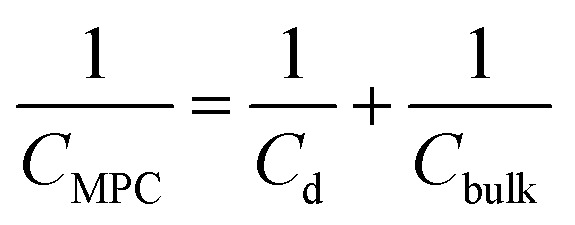
with

11 *C*_d_ = 4π*ε*_0_*ε*_d_*ε*_r_*r*(*r* + *d*)/*d*

12 *C*_bulk_ = 4π*ε*_0_*ε*_r_(*r* + *d*)

If we consider the ionic atmosphere around the NP, the bulk capacitance becomes^17^

13 *C*_bulk_ = 4π*ε*_0_*ε*_r_(*r* + *d*)[1 + *κ*(*r* + *d*)]

with *κ* the Debye length, determined by the ionic strength of the electrolyte solutions and the relative permittivity of the solvent. These simple equations illustrate the dominating effect of the monolayer when determining the values of the capacitance and, hence, the separation between the different redox potentials.

However, this equation does not include the effect of the charge on the capacitance of the NP, as linearized Poisson–Boltzmann equation was used to account for the electrochemical double layer.^17^ The most sophisticated approach involves a generalized P–B equation that considers the monolayer as a disordered medium where the thermal motion of the counter ions is decreased because of electrostatic correlations and monolayer structural effects. This generalised P–B equation was proposed on the basis of replacing the Boltzmann distribution by the Tsallis *q*-exponential distribution.^23^ The comparison of these models show that capacitance calculated from the simple concentric sphere model (eqn (11)) gives both the correct order of magnitude as well as some qualitative features of the total MPC capacitance, although significant deviations are predicted especially when ion and solvent penetration are important. Recent works by Su *et al.* have also stressed the importance of ion penetration into the monolayer.^24,25^

## The Fermi level and redox potential of a soluble metallic NP in solution

The link between *E*NPF and *r* of a metallic NP has also been highlighted by the pioneering theoretical and experimental work of Plieth^26^ and Henglein.^27^ Henglein predicted large shifts to higher *E*NPF as *r* decreased on the basis of gas phase thermodynamic data and kinetic measurements. These results revealed that for exceptionally small metallic NPs of silver,^27–29^ copper,^30^ lead^27^ and others with one to fifteen atoms present, the predicted negative shifts were not smoothly monotonic but oscillated due to small quantum mechanical effects at this scale. *E*NPF for Ag_*n*_ clusters is predicted to rise to such extents with decreasing *r* that for the smallest odd atom clusters, *n* = 1 and 3, the expected redox potentials are:^28^ 
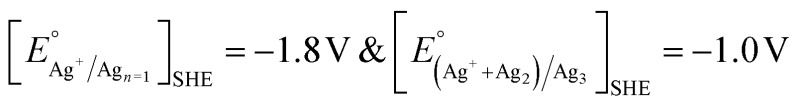
By comparison, the standard redox potentials of bulk silver and the strong reducing agent zinc are +0.799 V and –0.76 V, respectively. In fact according to eqn (2), the redox potential for the oxidation of a silver atom corresponds to the ion–solvent interaction energy of *ca.* –476 kJ mol^–1^ when considering the ionization energy of Ag atom of 731 kJ mol^–1^. This compares well with the hydration energy of –430 kJ mol^–1^.^31^

Plieth considered the contribution of the chemical potential of a metal atom on a metallic NP of the same metal for growth and dissolution reactions in the presence of reducing or oxidising agents, respectively. For the reduction of a metal cation resulting in the addition of a metal atom to the NP: M^+S^ + e^–V^ + NP_*n*_^*ze*^ ⇄ NP_*n*+1_^*ze*^, the standard redox potential differs from that on a large metal electrode by a term inversely proportional to *r*. As a first approximation considering only a polycrystalline NP we have:14
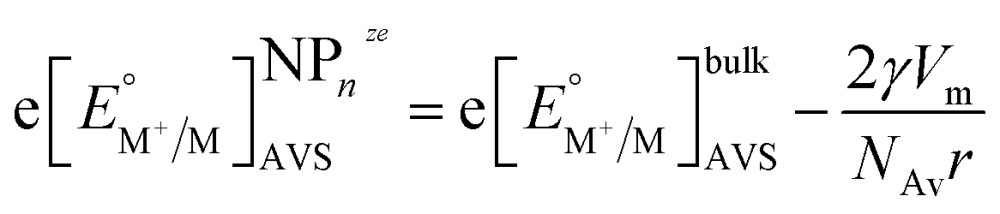
where *γ* is the surface tension, *N*_Av_ is Avogadro's constant and *V*_m_ is the molar volume of the metal. This approach considers the change in Gibbs free energy associated with an increase in the metals surface area, but it does not take into account the differences in surface energies of different facets. Additionally, surface energy depends also on the Galvani potential difference between the NP and the solution according to the Lippmann equation.^32^ The additional term in eqn (14) accounts for the difference of the chemical potential of a metal atom between a bulk metal and a NP. Eqn (14) is applicable for dissolution and growth reactions in solution containing metal cations and additional oxidising or reducing agents, but it does not consider the charge of the NPs.

 Eqn (8) and (14) account for different phenomena. Eqn (8) expresses the variation of the redox potential upon charging or discharging of a metallic NP capable of storing either positive or negative charges upon oxidation or reduction, respectively. Eqn (14), on the other hand, accounts for the size effect of the redox potential for (i) the reduction of a metal cation resulting in the growth of a NP or (ii) the oxidation of a metallic NP not capable of storing positive charges as it dissolves upon oxidation. The major difference between eqn (8) and (14) is that eqn (8) gives the Fermi level of the electron on the metallic NP whereas eqn (14) gives the Fermi level for the electron in solution for the redox couple M^+^/M^NP^. This is not often clear in the literature and often a source of confusion. Of course, at equilibrium eqn (8) and (14) should be equal, thereby defining a relationship between the excess charge and *r* of the NP according to this simple electrostatic model. This is illustrated in Fig. 1B for the case of gold with chloride. For example, 5 nm radius Au NP in equilibrium with AuCl_4_^–^ solution with an activity of 1 has a positive excess charge of +32*e*.

 Eqn (14) is a simple form of the more general equation15
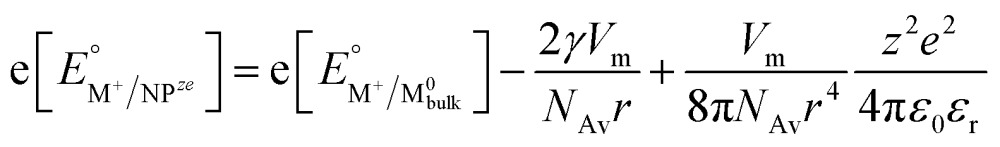
that also considers the charge of the metallic NP. This expression can be derived by utilizing the chemical potential of the charged NP presented by Lee *et al.* ^33^

The higher *E*NPF of some metallic clusters allows seemingly strange reactions not possible with bulk materials, for example electron transfer from a noble metal to a non-noble metal. Indeed, Henglein reported such a reaction involving electron transfer from small Ag clusters to Cu^2+^.^30^ Experimental proof of the predicted exceptional reducing abilities of small metallic clusters was found indirectly by investigating their abilities to reduce organic molecules.^27–29^

The difficulties in designing experiments that directly show *E*NPF rising, and the NP stability decreasing, with decreasing *r* is reflected in several seemingly contradictory reports from electrochemical stripping voltammetry,^34–37^ electrochemical scanning tunnelling microscopy (STM) and microscopy experiments.^38–41^ However, these contradictory results are on reflection not overly surprising as, though thermodynamically the stability of a NP should decrease with decreasing *r*, other interfering mechanisms may come into play that unexpectedly stabilize the NP. Such unpredictable stabilizing mechanisms are particularly a problem at the extreme nanoscale and this is exactly the size-regime where the most dramatic changes in *E*NPF and redox potentials are expected with decreasing *r*.

**Fig. 2:**
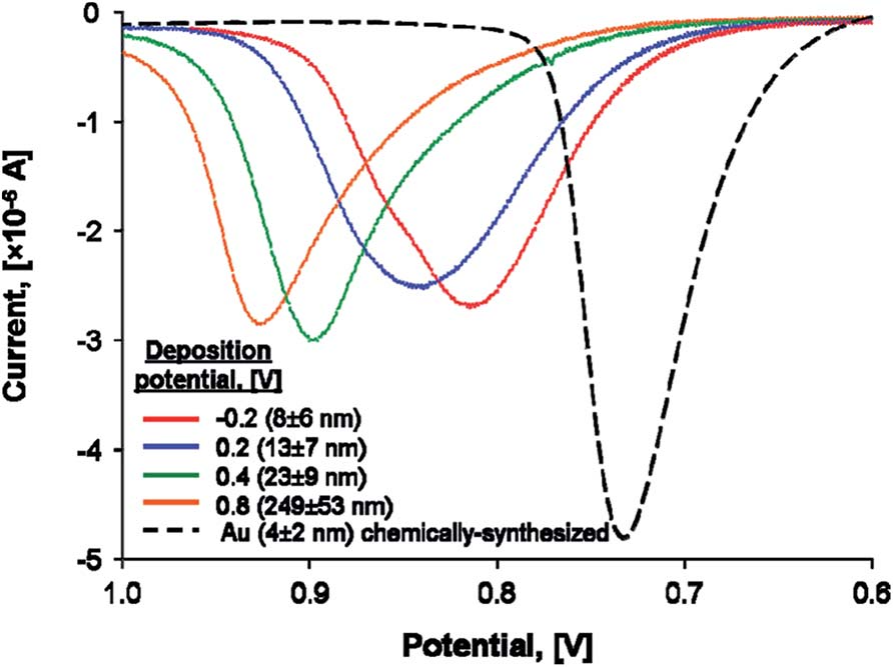
Experimentally observed shifts of the oxidative stripping peak potentials *E*_P_ with decreasing metallic NP radii. Linear sweep voltammograms (LSVs) were obtained in 10 mM potassium bromide plus 0.1 M HClO_4_ electrolyte at 1 mV s^–1^ on glass/ITO electrodes coated with electrochemically or chemically synthesized Au NPs of varying radii. Adapted with permission from ref. 36; O. S. Ivanova and F. P. Zamborini, *Anal. Chem.*, 2010, **82**, 5844–5850. Copyright 2010 American Chemical Society.

One seemingly simple and direct experimental approach to prove that a metallic NP becomes less stable as *r* decreases is to attach it to a conductive electrode surface and carry out stripping voltammetry. The position of the peak potential, *E*_P_, for the stripping peak represents the oxidative dissolution of the metallic NP to ions and is a direct indication of the NPs stability. According to Plieth,^26^ Henglein,^27,29,30^ and eqn (8) and (14) above, one would logically expect to see *E*_P_ shift negatively (easier to oxidise) as *r* decreases (Fig. 2).

Indeed, this approach has been utilized by the groups of Compton^34^ and Zamborini.^35–37^ Zamborini *et al.* observed *E*_P_ of chemically synthesized Ag NPs attached to indium tin oxide (ITO) electrodes by amine linker molecules shifting negatively as a function of *r* for NPs < 35 nm in size.^35^ The latter results are qualitatively in agreement with the thermodynamic predictions, however, little variation was observed in the 35–50 nm size range.^35^ These authors also investigated the stripping of electrodeposited 4–250 nm Au NPs^36^ and chemically synthesized and tethered Au NPs with sizes <4 nm (ref. 37) in the presence of halides at conductive ITO electrode surfaces (see Fig. 2). In both instances, *E*_P_ shifted negatively with decreasing *r*, once more in qualitative agreement with the thermodynamic expectations. The dissolution of Au NPs is much more complex than that of Ag NPs, however, requiring considerable further study to elucidate the precise mechanism of Au oxidation and complexation.

The dissolution of small clusters of atoms or NPs on conductive substrates has also been monitored by STM and microscopy. Sieradzki *et al.* observed the oxidative dissolution of <4 nm Pt NPs at potentials less than the bulk potential.^41,42^ Furthermore, they made a distinction between the mechanisms of Pt dissolution between the NP and the bulk metal. Whereas bulk Pt dissolves from the oxide, the Pt NPs are dissolved by a direct electrochemical route involving the electro-oxidation of Pt NPs to Pt^2+^ ions.^41,42^ Similarly, Del Popolo *et al.* reported that small Pd NPs dissolve at more negative oxidation potentials relative to bulk Pd.^40^ Meanwhile, Penner and co-workers electrodeposited Ag NPs on HOPG and by microscopy noted a thermodynamically unexpected enhanced stability of the NPs in comparison to the bulk Ag.^39^ Currently, there is considerable debate in the literature to explain these contradictory results. One possibility is that the interaction of the electrode surface with small NPs has stabilizing effects, possibly due to mechanical alloying or quantum mechanical effects that render the bulk energy term inappropriate to describe the bonding.^38^ Sieradzki *et al.* put forward several other possibilities including the NPs compensating their increased energy by bonding more strongly with passivating agents in solution, such as oxygen, protons, or hydroxyl groups.^41,42^ They also attribute the primary source of error in the thermodynamic predictions to the use of bulk surface and cohesive energies and the neglect of edge and vertex atoms in NPs.^41,42^

Another approach by Miaozhi *et al.* demonstrated the effect of the oxide particle size on the equilibrium potential between bulk silver and Ag_2_O NPs.^43^ The reaction is:Ag_2_O + H_2_O + 2e^–^ ⇌ 2Ag + 2OH^–^.By considering the chemical potential of Ag_2_O NPs, they obtain16
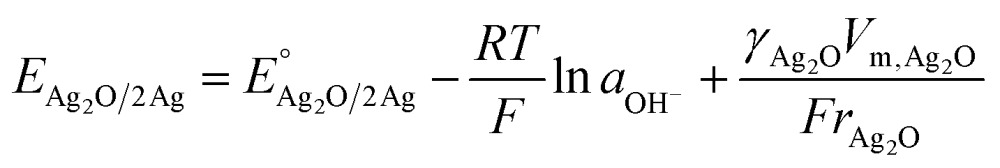
In this case, the decreasing cluster size increases the redox potential. This approach allows the surface energy of the NPs to be determined. For Ag_2_O, the surface energy can be estimated from the slope of the redox potential *vs.* 1/*r*. An experimental slope of 1.283 V nm gives a value of 0.381 mJ cm^–2^.

## Electrochemical equilibria between metallic NPs and species in solution or electrodes

### Electrochemical equilibria in solution

**Scheme 3:**
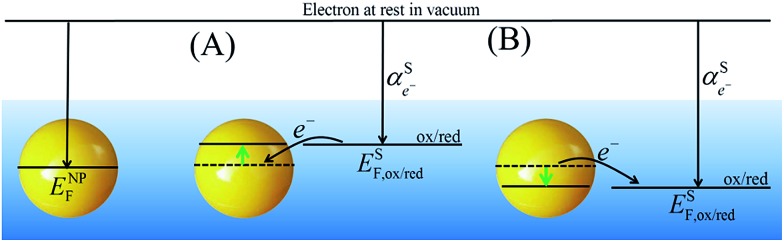
Redox equilibria for metallic NPs in solution showing the capabilities of metallic NPs to be (A) charged and (B) discharged upon Fermi level equilibration with an excess of a single dominant redox couple in solution.

**Fig. 3:**
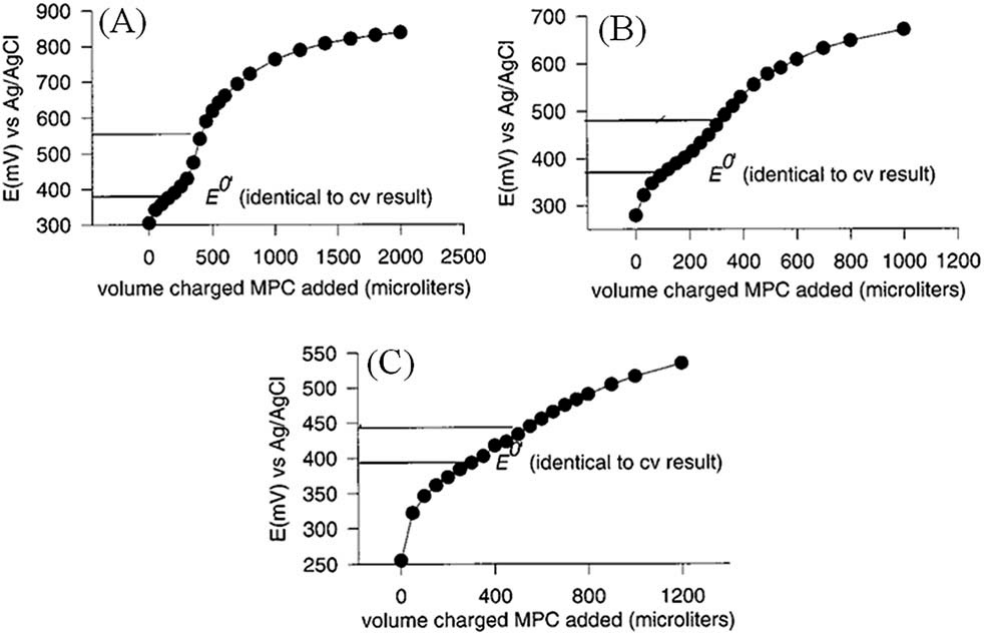
Potentiometric titrations of charged Au MPCs with an electron donor, ethylferrocene. Incremental additions of 0.30 mM Au MPC solutions, charged by electrolysis to (A) +0.92 V, (B) +0.72 V and (C) +0.62 V (*vs.* Ag/AgCl), respectively, were made to 5 mL solutions of ethylferrocene in (2 : 1) toluene–acetonitrile mixtures with 0.1 M tetrabutylammonium hexafluorophosphate supporting electrolyte. The concentration of ethylferrocene was 76 μM in (A) and 36 μM in (B) and (C). Figure is adapted with permission from ref. 44; J. J. Pietron, J. F. Hicks and R. W. Murray, *J. Am. Chem. Soc.*, 1999, **121**, 5565–5570. Copyright 1999 American Chemical Society.

It is important to realise that a metallic NP immersed in a solution will reach, albeit sometimes very slowly (is some cases this might take days or even years), an electrochemical equilibrium with the surrounding solution. If the redox potential in solution is dominated by a single redox couple, ox/red, in excess, then the Fermi level of the electrons in the metallic NP, *E*NPF, will change to become equal to the Fermi level of the electrons in solution, *E*SF,ox/red, for this redox couple. This change results in either an electrostatic charging of the metallic NP accompanied by an oxidation of the redox couple in solution or discharging of the metallic NPs accompanied by a reduction of the redox couple in solution. Both scenarios are illustrated in Scheme 3. In this case, it is assumed that the metallic NP itself is completely chemically inert in solution. Under standard conditions (*c*_ox_ = *c*_red_), this equilibrium is given by17
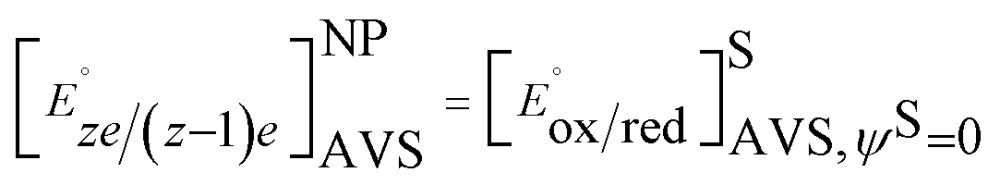
In other words, the charge on the metallic NPs will be imposed by the redox couple in solution to satisfy eqn (17). This has been proven experimentally by performing potentiometric titrations of NPs, demonstrating that NPs can behave as any normal redox couple (also see Fig. 3).^44^

At this stage, it is necessary to recognize that the initial potential of *E*NPF “at rest” immediately after synthesis is determined by the synthesis method chosen, and especially the stabilizing ligands employed, as well as the storage conditions, *i.e.*, aerobic or anaerobic. Thus, one could in principle view NP synthesis as a dynamic process that initially leads to the formation of metallic nuclei, followed by their growth until Fermi level equilibration of all of the components in the synthesis media (metallic ions, metallic NPs, reductant, solvent, oxygen, *etc.*) takes place. As a result, NPs prepared with a relatively weak reductant such as citrate have a lower *E*NPF than NPs prepared with a stronger reductant such as sodium borohydride (NaBH_4_).^45^ This residual charge remaining on the NP due to the reduction step is difficult to reproduce, and not always observed,^46^ but in theory will always be present unless a reductant with an identical reduction potential to *E*_F_ of the uncharged polycrystalline metal is employed.^45^ Thus, it is immediately apparent that Fermi level equilibration in metallic NPs is a crucial process that offers an alternative perspective not only on the behaviour and reactivity of metallic NPs, as discussed above, but also on their synthesis.

Once synthesized, *E*NPF of metallic NPs in solution is determined by their redox environment. Therefore, introduction of chemical reductants, such as NaBH_4_ or ascorbic acid,^47^ or oxidants, such as cerium(iv) sulfate (Ce(SO_4_)_2_),^46^ to the NP solution will result in *E*NPF raising or lowering, respectively, as depicted in Scheme 3. The latter is also true for biphasic systems as demonstrated by Wuelfing *et al.*, who used a biphasic approach to lower the Fermi levels of MPCs of Au suspended in dichloromethane by liquid–liquid interfacial electron transfer with an aqueous Ce(SO_4_)_2_ solution.^46^

### Fermi level equilibration of metallic NPs with polarized electrodes

**Scheme 4:**
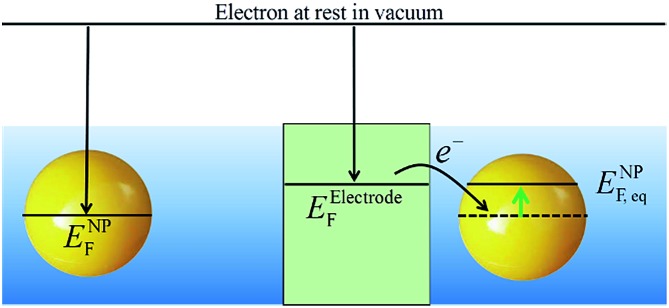
Equilibration of the Fermi level of the metallic NP with a polarized electrode.

*E* NP F may be raised or lowered by collision with a polarized electrode. Upon collision, the Fermi levels of the metallic NP and the electrode will equilibrate. As the Fermi level of the electrode is controlled by the voltage source, the Fermi level of the NP is shifted to reach the Fermi level of the electrode. Ung *et al.* demonstrated that *E*NPF of polymer-stabilized Ag NPs equilibrates with a polarized gold-mesh bulk electrode in solution by spectro-electrochemically monitoring the optical properties of the colloidal solutions upon charge–discharge (discussed *vide infra*).^48^ Due to the high ionic strength of the media, the NPs could theoretically (from DLVO theory) approach the electrode surfaces to within 1 or 2 nm and Fermi level equilibration was proposed to occur *via* tunnelling of electrons in their hundreds and thousands across the double-layers of the NP and electrode.^48^ Much earlier Miller *et al.* demonstrated that *E*NPF of Pt NPs could equilibrate with a reductively polarised Hg-pool working electrode using a methylviologen redox shuttle as a mediator.^49^ The equilibration of the Fermi level of the NP with the electrode is shown schematically in Scheme 4.

Pietron *et al.* ^44^ took advantage of the unique properties of MPCs of Au, namely the discrete quantized nature of their capacitive charging, to introduce a more quantitative approach to raising *E*NPF for a solution of Au MPCs. Stirred solutions of Au MPCs were subjected to classical electrolysis conditions at either oxidizing or reducing potentials, in a toluene–acetonitrile solvent. The Fermi levels of the Au MPC cores and electrode equilibrated by the injection or removal of electrons from the Au MPC core and the simultaneous formation of an ionic space charge layer around the Au MPC. The so-called quantized charging behaviour which in electrochemical terms would be called an oxidation or reduction reaction, monitored by differential pulse voltammetry, was used as a means to estimate the average “stoichiometric oxidizing or reducing capacity”, *i.e.*, the oxidizing (hole) or reducing (electron) equivalents per mole, of the Au MPCs in solution at an arbitrarily set potential. The resulting Au MPC solutions were shown to be both remarkably stable, discharging at very slow rates, and capable of maintaining their new oxidative or reductive potentials even after isolation in a dried form and re-dissolution in a new solvent. Potentiometric titrations of charged Au MPCs with electron donor and acceptor molecules, such as ethylferrocene and tetracyanoquinodimethane (TCNQ), respectively, revealed the ability of these nanoscopic metallic NPs to act as non-molecular oxidizing or reducing agents, see Fig. 3. The classical behaviour of these potentiometric titrations, proceeding in a predictable and quantifiable way, highlighted the ability of the Au MPCs to act as quantifiable electron or hole carriers by Fermi level equilibration with a polarised electrode.^44^

### Chemisorption of nucleophiles or electrophiles to metallic NPs

*E* NP F may be raised or lowered by the chemisorption of nucleophiles or electrophiles, respectively, at the NPs coordinatively unsaturated surface atoms.^30,50^ Such adsorbates can substantially influence the reactivity and optical properties (discussed *vide infra*) of the metallic NPs. The shift of *E*NPF can be due to (i) the charge of the nucleophile, for example anions, as discussed above in relation to eqn (8), charging the NP with adsorbed negative charges and raising *E*NPF but also (ii) the change of the surface potential of the NP, as shown in the case of neutral nucleophiles such as triphenylphosphine on silver.

**Scheme 5:**
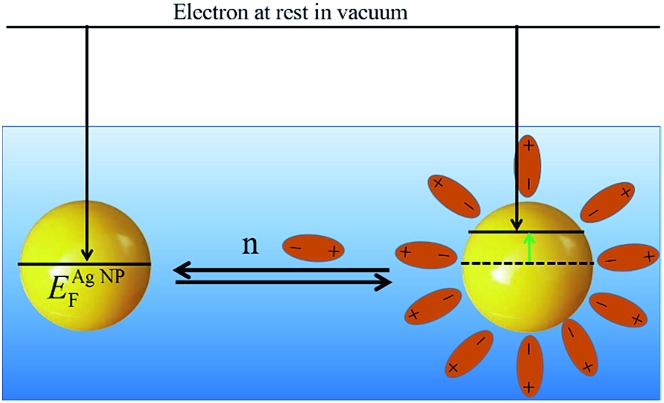
The Fermi level of a metallic NP is raised on chemisorption of neutral dipoles, modifying the surface potential and hence the Fermi level. The direction of change depends on the overall change in the surface potential. In this scheme the surface potential is lowered, making extraction of electrons easier.

As proposed by Henglein, a surface atom carrying a nucleophile will acquire a slight positive charge (*δ* ^+^) while excess electron density is transferred to the interior of the NP, which becomes slightly negative (*δ* ^–^).^50^ In this case, the nucleophile affects the local charge density of the surface, altering the surface potential and hence also the Fermi level. The effect of adsorbed species on surface potential is well known from for example work function measurements in UHV. For all nucleophiles, the surface potential *χ* and hence the real potential *α* of the NP are altered and the Fermi level changes according to the sign of the surface potential until equilibrium is reached (see Scheme 5). At this point, no further nucleophiles may be adsorbed and, in essence, this signifies that the nucleophile desorption/adsorption equilibrium is dictated by the position of *E*NPF. Consequently, by manipulation of *E*NPF the nucleophile desorption/adsorption equilibrium can be drastically shifted in either direction.

Full desorption of nucleophiles can be achieved by adding or subtracting excess electrons to the NP. This behaviour is similar to ion or molecule adsorption on polarized metal electrodes, where surface coverage of the electrode can be adjusted by changing its potential (Fermi level). One approach is to introduce highly reducing free radicals to the solution and the full desorption of anionic nucleophiles such as hydrogen sulfide (SH^–^) and iodide (I^–^) anions from the surface of Ag NPs was achieved in this manner.^51^ Another approach is to raise *E*NPF by chemisorption of a highly reducing nucleophile that prevents the initial adsorption or induces the full desorption of another competing nucleophile. Indeed, in such a manner, SH^–^ is capable of fully desorbing I^–^ from the surface of a Ag NP under conditions where the surface of the NP is unsaturated with SH^–^ (*i.e.*, sub-monolayer conditions).^51^

The adsorption of nucleophiles, and the subsequently raised *E*NPF, renders metallic NPs drastically more susceptible to oxidation and therefore dissolution. The equilibrium may be shifted to favour further nucleophile adsorption by discharging the metallic NP with O_2_ or weak electron acceptors that ordinarily would not react with the metallic NP in the absence of chemisorbed nucleophiles. For instance, Ag NPs may be oxidized and dissolved by weak electron acceptors such as nitrobenzene or MV^2+^ that would ordinarily never attack them.^50^ Nucleophilic CN^–^ and SH^–^ chemisorbed to Pd NPs raise *E*NPF to such an extent that the Pd NPs begin to dissolve forming Pd(CN)_4_^2–^ and PdS, respectively, in the absence of an oxidant by reducing water and producing H_2_.^52^ Additionally, as a metallic NP dissolves, *r* decreases and *E*NPF increases (eqn (14)), thus further increasing the driving force for dissolution.

**Fig. 4:**
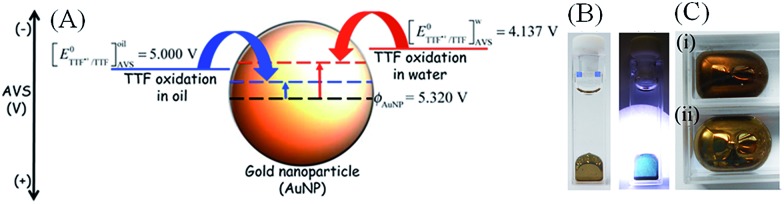
Ligand displacement with nucleophiles in solution facilitated by Fermi level equilibration. Reflective gold metal liquid-like droplets (MeLLDs) were formed by emulsification of 1 mL of 1,2-dichloroethane solution containing 1 mM tetrathiafulvalene (TTF) with certain volumes of citrate-stabilised colloidal AuNP solutions, followed by some time to allow the emulsion to separate on settling. TFF adsorbs on the surface of the Au NP, acts as a nucleophile and undergoes Fermi level equilibration with the Au NP becoming oxidized to TTF˙^+^. The resulting increase in *E*NPF may be a factor in the electrostatic displacement of citrate from the surface of the Au NP, replaced by adsorbed TTF˙^+^. (A) Schematic of the shift in the Fermi level of the Au NPs during charge (electron) transfer with TTF by Fermi level equilibration. (B) The resultant gold MeLLDs looks like gold droplets when viewed in reflection mode for 76 nm diameter Au NPs (on the left) and act as blue filters in transmission mode with smaller 14 nm diameter Au NPs (on the right). The colour reflected by the Au MeLLDs differs depending on the size of the Au NPs used, with (C) (i) smaller 14 nm diameter Au NPs giving reddish-brown films and (B) (ii) large 76 nm diameter Au NPs producing films that resemble bulk gold. Adapted with permission from ref. 53; E. Smirnov, M. D. Scanlon, D. Momotenko, H. Vrubel, M. A. Méndez, P.-F. Brevet and H. H. Girault, *ACS Nano*, 2014, **8**, 9471–9481. Copyright 2014 American Chemical Society.

Recently, Smirnov *et al.* introduced a facile method to encapsulate oil droplets in an unbroken film of Au NPs, creating “metal liquid-like droplets”.^53^ In this study, aqueous citrate stabilized Au NPs were emulsified with an organic solution of 1,2-dichloroethane containing a lipophilic electron donor, tetrathiafulvalene (TTF, see Fig. 4). After emulsification and settling, the aqueous phase became devoid of any Au NPs with a lustrous gold film now present at the liquid–liquid interface. The suggested mechanism involves TTF acting as a nucleophile injecting electrons into the Au NP, raising *E*NPF until the system reaches Fermi-level equilibration and, thereby, significantly influencing the adsorption/desorption dynamics of citrate and TTF species. Specifically, at equilibrium, the more reduced Au NPs may induce the removal of anionic citrate ligands electrostatically, further facilitating the absorption of TTF˙^+^. The removal of the electrostatically stabilising citrate ligands ultimately causes the Au NPs to aggregate and form a dense film at the liquid–liquid interface. Such a mechanism was supported by the observations of Weitz *et al.* who noted that TCNQ, adsorbed as its radical anion, TCNQ˙^–^, cannot displace citrate.^54^ As an electron acceptor or electrophile, TCNQ lowers *E*NPF during charge transfer to a less reducing (*i.e.* more positive) potential, thereby increasing the electrostatic attraction between citrate and the surface of the Au NPs.

### Metallic NPs landing experiments

**Scheme 6:**
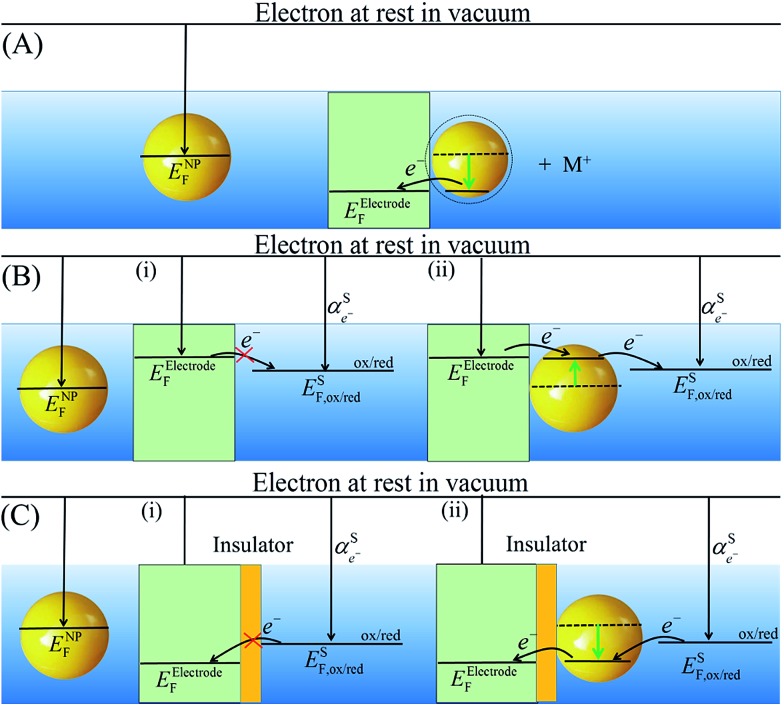
Fermi level equilibration between polarized electrodes and metallic NPs that “land” or “impact” on the electrode surface. (A) Oxidative dissolution of NPs by lowering their Fermi levels on impact with a positively polarized electrode. (B) “Electrocatalytic amplification” of metallic NP impacts by raising *E*NPF on impact and thereby electrocatalyzing a reduction reaction that is kinetically limited on the electrode surface. (C) The NP enhanced tunnelling of electrons through the insulating layer, lowering *E*NPF on impact and thereby electrocatalyzing an oxidation reaction that does not happen without the NP.

Over the past twenty years, a new area of research based on Fermi level equilibration between NPs and ultramicroelectrode (UME) surfaces known as “nanoparticle impact” or “nanoparticle landing” electrochemistry has developed at pace.^55^ The premise of this field is that when NPs, travelling under thermal Brownian motion in an inert electrolyte solution, strike a polarized UME surface (*e.g.*, a carbon fibre or a mercury-plated Pt microelectrode) the impacts are directly detected electrochemically as sharp current–time transients at a constant electrode potential^55^ or potentiometrically by monitored changes of the open-circuit potential.^56^ The simplest experiments involve direct electrochemical measurements of the NPs by raising and lowering of *E*NPF on impact. The majority of these studies involve oxidation (and subsequent dissolution, see Scheme 6A) of Ag,^57^ Au,^58^ Cu^59^ or Ni^60^ NPs to reveal a host of information such as the NP size distributions and conductivities,^57^ identification of individual NPs in a mixture,^61^ the concentrations of the NPs,^61^ the NP residence time on the electrode sufficient to ensure complete oxidation,^62^ *etc.* Bard *et al.* demonstrated that single NP collisions with an electrode could be observed *via* electrocatalytic amplification.^63^ In effect, on contact with a polarized substrate, the NP acts as a single nanoscopic spherical electrode for redox reactions that are catalysed only on the NP and not the underlying electrode.

**Fig. 5:**
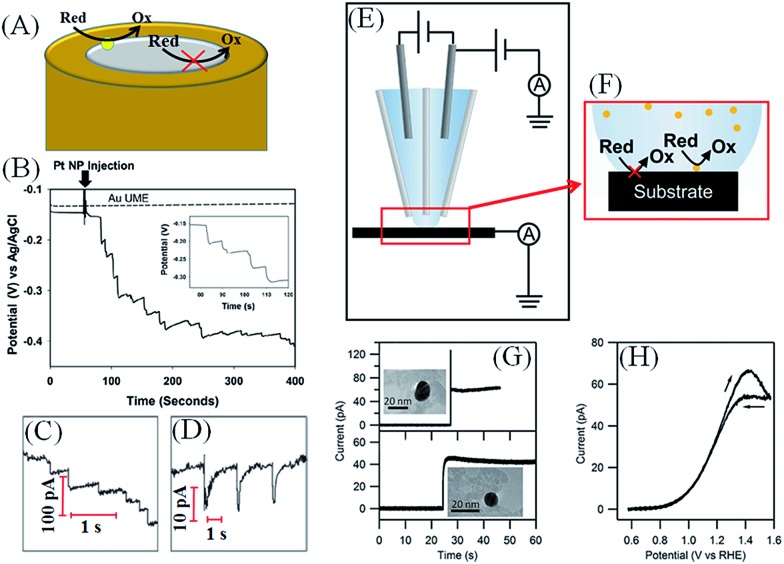
(A) NP impact or landing studies involve polarizing an inert ultramicroelectrode (UME) in a solution containing a reactant (ox/red) and catalyst NPs (yellow sphere). As the NPs impact the surface they undergo Fermi level equilibration with the UME, as described in Scheme 6, convert the reactant to product and generate a measurable current. (B) Potentiometric monitoring of the open circuit potential (OCP) *vs.* time for single Pt NP collisions at the surface of a 5 μm radius Au UME in the presence of 5 mM phosphate buffer (pH 7.0) and 15 mM hydrazine with (solid line) and without (dashed line) 7.5 pM Pt NPs. Inset: magnified image of the staircase potential response. Alternatively, current transients may be detected and exhibited. Adapted with permission from ref. 56; H. Zhou, J. H. Park, F.-R. F. Fan and A. J. Bard, *J. Am. Chem. Soc.*, 2012, **134**, 13212–13215. Copyright 2012 American Chemical Society. (C) A “staircase” response, if the NPs “stick” to the UME generating a continuous response, as seen for hydrazine oxidation by Pt NPs impacting a polarized Au microelectrode, or (D) a “spike” or “blip” response, as seen for water oxidation by IrO_*x*_ NPs impacting a Pt microelectrode. (C and D) reproduced from ref. 71 with permission from the PCCP Owner Societies (E) schematic representation of a scanning electrochemical cell microscopy (SECCM) set-up as described by Unwin *et al.* and (F) a schematic of the liquid meniscus that constitutes the electrochemical cell. Analogous to conventional NP impact studies, the electrode substrate is held at a potential where a reaction occurs on the catalytic impacting NPs but not on the collector electrode. (G) Landing events of individual Au NPs using the SECCM setup with the same Au NP imaged by TEM afterwards providing exceptional opportunities for structure-electrochemical reactivity analysis. (H) Cyclic voltammogram for the oxidation of 2 mM hydrazine measured at a scan rate of 200 mV s^–1^ for the individual AuNP shown in (G) on a TEM grid electrode. (E–H) adapted with permission from ref. 70; S. E. F. Kleijn, S. C. S. Lai, T. S. Miller, A. I. Yanson, M. T. M. Koper and P. R. Unwin, *J. Am. Chem. Soc.*, 2012, **134**, 18558–18561. Copyright 2012 American Chemical Society.

For example, by rising *E*NPF of Pt NPs on impact with a carbon UME, the NPs were able to act as nanoelectrodes and catalyse proton reduction (see Scheme 6B).^63,64^ Similarly, lowering *E*NPF on impact allowed Pt NPs colliding with a Au UME to oxidize hydrazine^64^ and IrO_*x*_ NPs colliding with a Pt UME (pre-treated with NaBH_4_) to oxidize water.^65^ Much work has focused on mechanism elucidation and developing a clear appreciation of how the NP interaction with the electrode, in terms of NP residence time,^65^ permanent NP adsorption^64^ and NP deactivation,^66^ or lack thereof,^67^ after adsorption, significantly affects the observed potentiometric responses, see Fig. 5B, or current signals (permanent stepwise changes, see Fig. 5C, *vs.* transient spikes, see Fig. 5D). Finally, core–shell metallic NPs may be synthesized by NP collisions at a reductively polarized electrode in the presence of the ions of another metal. Using this approach, by raising *E*NPF of Ag NPs on impact, Tl^+^ was deposited to form Ag@Tl NPs,^68^ while Cd^2+^ was deposited to form Ag@Cd NPs.^69^

Unwin and co-workers^70^ recently introduced a novel method to carry out landing experiments using scanning electrochemical cell microscopy (SECCM, see Fig. 5E and F). Their approach involves miniaturising the “landing area” by using tiny electrochemical cell volumes and not tiny electrodes. Thus, landing experiments take place on a polarised macro-sized electrode in contact with a nanodroplet of solution, rather than the typical approach of immersing an UME in a large volume of solution.

In a proof of concept paper, SECCM was used to show the O_2_ reduction reaction (ORR) and HER at the surface of impacting Au NPs by modulating *E*NPF on changing the potential at the HOPG surface.^70^ Uniquely, this approach allows landing experiments on substrates that are not amenable to UME manufacture such as HOPG and even a carbon-coated TEM grid. Strikingly, the ability to electrochemically interrogate individual NPs on a TEM grid means that unambiguous correlations of a single NPs physical attributes and electrochemical properties, *e.g.* catalytic activity, are possible (see Fig. 5G and H).^70^

Another interesting property of metallic NPs is described in Scheme 6C. NPs can significantly enhance tunnelling of electrons through an insulating layer (either self-assembled monolayer or solid layer).^71,72^ Tunnelling to the metallic NP is much more probable than tunnelling to molecules in solution, as NPs have significantly higher density of states compared to dilute molecular redox species in solution.^73^ Tunnelling currents decay exponentially with *i* ∝ *A* exp(–*βd*) over distance *d*, where parameter *β* depends only on the insulating layer. However, a higher density of states significantly enhances the pre-exponential factor,^74^ allowing tunnelling to metallic NPs when tunnelling to molecules is no longer possible. This phenomena has been utilized to fabricate robust and stable tunnelling nanoelectrodes, where a single metallic NP (acting as the electrode) is captured in a collision with an insulating layer on an UME.^73^

## Experimental approaches to quantifying changes in Fermi levels after charge and discharge events

Quantitative comparison of the relative apparent Fermi levels of differently functionalized floating supported NPs may be achieved by titrating the stored electrons in charged metallic NPs and also NP-semiconductor (SC) nanocomposites with electron acceptor molecules (A) such as C_60_ (ref. 75) and dyes (*e.g.*, methylene blue^76,77^ or thionine^76,78^). Such an approach was used by Grätzel^79^ and Nozik^80^ in the 1980's, to make a connection between the energy level of electrolyte redox molecules and those of SCs, and has since found prominence with the group of Kamat to determine the electrons stored in SCs and their nanocomposites with both metallic NPs^78^ and carbon supports.^76^ The key to these measurements is that the standard reduction potential of A, 
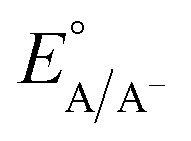
, in the solvent containing the nanocomposites is known and used as a reference to calibrate the apparent Fermi level *vs.* SHE. The Fermi level of the nanocomposite establishes a Nernstian equilibrium with the A/A^–^ redox couple, [A^–^] and [A] are determined by UV/vis spectroscopy and the Nernst equation is applied to determine the apparent Fermi level, E*F.18
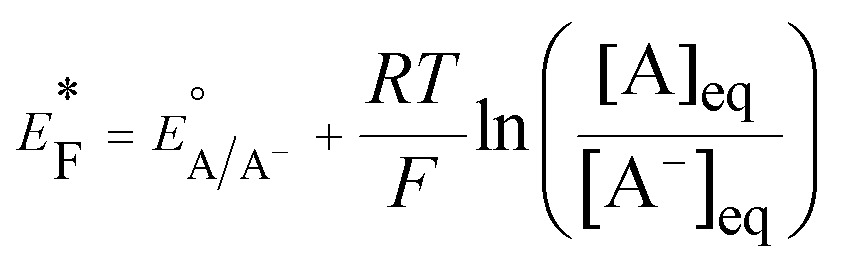
The yield of A^–^ will increase as 
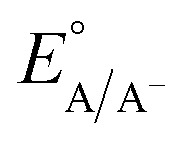
 shifts anodically to more positive potentials. Thus, this technique provides information on the relative differences in apparent Fermi levels between two different nanocomposites by titrating identical quantities of both with the same redox probe. A relatively lower yield of A^–^ for a particular nanocomposite indicates that it has a more positive Fermi level and provides less driving force for electron transfer.

A number of alternative techniques exist that allow quantitative monitoring of changes in the Fermi level of supported metallic NPs due to charge injection and discharge. For example, an estimation of the number of electrons stored on either charged carbon (C) or SC supports is possible by discharging their stored electrons to reduce metallic ions, such as Ag^+^, in solution to form Ag/C,^81^ Ag/SC^77^ or even Ag/C/SC^82^ nanocomposites. Although such an approach can quantify the number of electrons stored on the nanocomposite, and provides a useful method to prepare metallic NP nanocomposites, it does not provide information on the apparent Fermi level of the materials as it is unreferenced (unlike potentiometry of Au MPCs which is referenced to a reference electrode in the electrochemical cell or the titration method for large metallic NP/SC nanocomposites, described above, which is referenced to *E* ^o^). In the specific case of ZnO, the injection and storage of electrons into the conduction band may be monitored by spectroscopy as charge storage causes the absorption band edge to shift to lower wavelengths and, simultaneously, the green emission arising from oxygen vacancy to disappear.^78,82,83^ Thus, for ohmic materials such as Pt NPs deposited on ZnO an almost complete recovery of the emission is seen, as the Pt NPs efficiently discharge electrons to the solvent, whereas with Au NPs only 60% of the emission recovered as a portion of the electrons remain on the ZnO due to the capacitive storage of electrons on Au and their poor discharge abilities.^78^

**Fig. 6:**
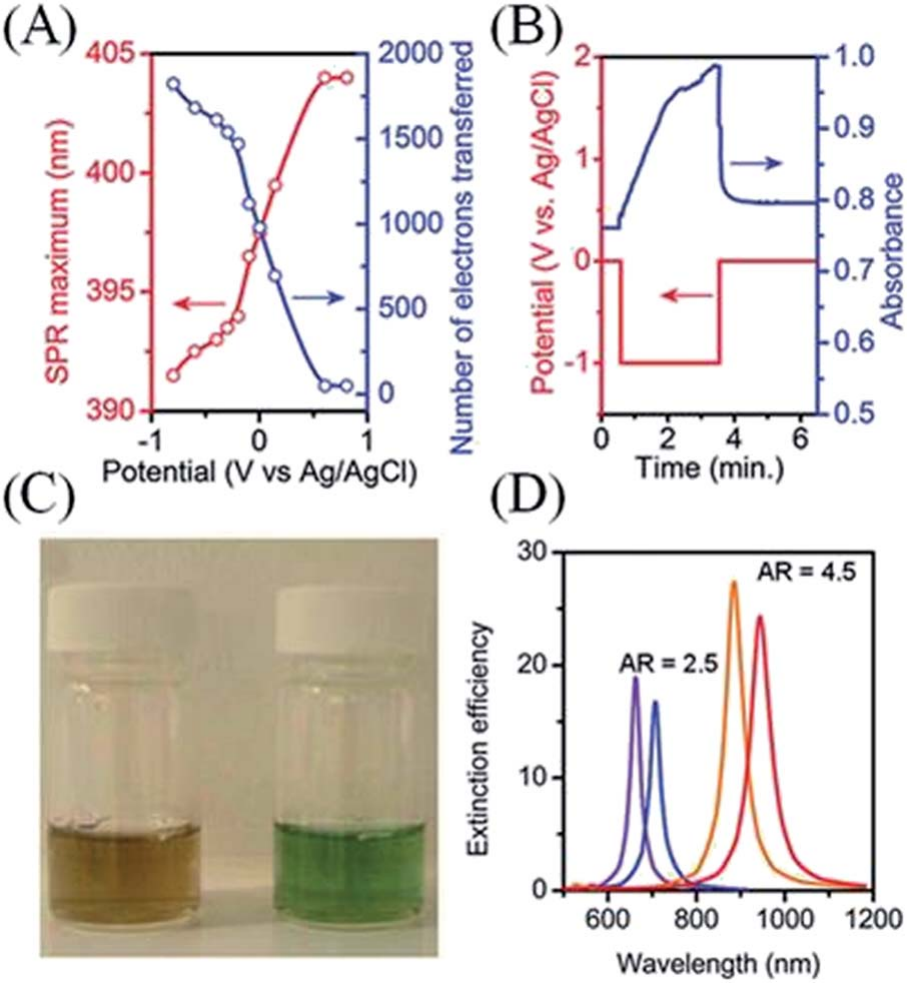
Surface plasmon spectroscopy (SPS): effect of applied potential and metallic NP shape on the shift of Fermi level as monitored by changes in surface plasmon resonance (SPR) for metallic NPs. (A) The Fermi level of Ag NPs was equilibrated over 300 s with a variety of applied potentials at a gold mesh electrode across a 2 V window. The latter shifts in Fermi level in turn induced a shift of ∼13 nm in the SPR across the potential range. This shift was used to estimate the number of electrons transferred to each ∼10 nm Ag NP. (A), (B) and (D) adapted with permission from ref. 84; S. C. Warren, D. A. Walker and B. A. Grzybowski, *Langmuir*, 2012, **28**, 9093–9102. Copyright 2012 American Chemical Society. Original data from ref. 48. (B) Spectral shifts at 393 nm as a function of time as monitored for an ITO electrode coated with a polymer layer containing Ag NPs as the potential was scanned to progressively more negative values. Original data from ref. 85 re-graphed by Warren *et al.* ^84^ (C) Photograph of vials containing nanorods before (left) and after (right) the addition of sodium borohydride, which raises the Fermi level of the nanorods by injecting electrons (see Scheme 3(A)) and thereby induces a blue-shift in the SPR. Reproduced with kind permission from Springer Science and Business Media from ref. 47; *Plasmonics*, **1**, 2006, 63, Drastic Surface Plasmon Mode Shifts in Gold Nanorods Due to Electron Charging, P. Mulvaney, J. Pérez-Juste, M. Giersig, L. M. Liz-Marzán and C. Pecharromán, Fig. 2, Copyright 2006 Springer. Original caption “Photograph of a CTAB-stabilized 0.25 mM gold nanorods solution in water with an aspect ratio of 3.7 before and after addition of 0.01 M NaBH_4_”. (D) Calculated shifts in the longitudinal surface plasmon resonance (LSPR) for gold nanorods of two different aspect ratios (2.5 : 1 and 4.5 : 1) upon a change in electron density of 14%. Plots generated by Warren *et al.* ^84^ using data taken from ref. 86.

Pioneering work by Henglein and Mulvaney highlighted that changes in the Fermi level of metallic NPs due to charging or discharging events directly influence their localized surface plasmons.^45,48,85^ A technique based on this principle, surface plasmon spectroscopy (SPS), monitors changes in the Fermi levels of metallic NPs that exhibit well-defined surface plasmon modes (Fig. 6). The relationship between the surface plasmon resonance (SPR) wavelength maximum (*λ*) and the relative electron concentrations (*n*) before and after electron injection or discharge is given by^84^
19
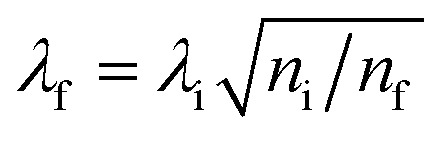
Thus, charging of metallic NPs leads to a blue-shift of the SPR, while discharging leads to a red-shift (Fig. 6A and B). The magnitude of the shift in SPR is highly dependent on the aspect ratio of the metallic nanorods (Fig. 6C and D). Greater changes in wavelength (Δ*λ*) are observed for the same change in electron concentration (Δ*n*) for metallic nanorods *vs.* nanospheres and for high-aspect *vs.* low-aspect nanorods.^47^ Mulvaney *et al.* have furthermore defined the relationship that links the SPR to the nanorods shape and the shift of the Fermi level with *n* as^47^
20
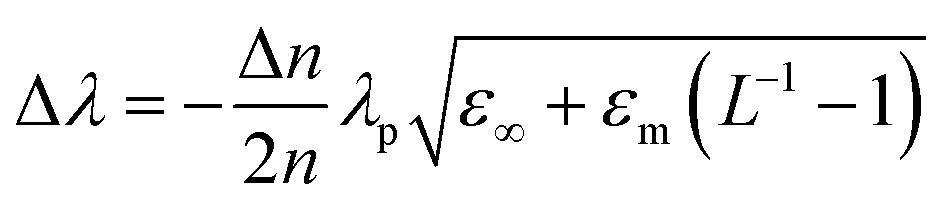
where *λ*_p_ is the bulk plasma resonance, *ε*_∞_ is the high-frequency contribution from interband transitions, *ε*_m_ is the dielectric function of the medium and *L* is the shape-dependent depolarization factor.

The versatility of SPS lies in its ability to probe any event that leads to a change in a metallic NPs Fermi level, such as chemisorption,^45,47,51,87^ core–shell formation by underpotential deposition of metals,^88,89^ Fermi level equilibration between metallic NPs and SCs in NP/SC^77,83^ or core–shell NP@SC^90^ nanocomposites, electron transfer from solution phase reductants and oxidants^47^ or by interaction with polarized electrodes,^48,85^ *etc.* The current state-of-the-art in SPS seeks to use the technique to study redox reactions at single metallic NPs.^16,91^ The transition from studying ensembles of NPs to single NPs will allow the effects of NP size, shape and interaction with the substrate on the rates of catalysis to be precisely determined instead of being obscured by incorporation into ensemble averages.^16,91^ As SPS is a non-invasive optical technique that is influenced by the redox environment within which the metallic NP finds itself, much future work is envisioned where SPS will indirectly relate the redox conditions inside biological tissues and cells optically.

## Perspectives

Metallic NPs are increasingly becoming part of everyday consumer products, ranging from cosmetics to clothes to medical and electrical devices. Thus, it is inevitable that our exposure to these nanomaterials will increase, as they appear in air, water, soil and organisms due to the ramp up in metallic NP production to meet consumer demand.^92,93^ The attractive features of Ag NPs that make them commercially sought-after, in particular their antimicrobial effects, on the flip-side cause them to be greatly detrimental to many mammalian organs.^94^ Thus, it is now an imperative grand-challenge for the nanotechnology community to (i) thoroughly investigate the nanotoxicity of metallic NPs to humans, animal and plant life under various environmental conditions^95^ and (ii) develop sensitive and selective analytical methods to detect and determine the environmental fates of metallic NPs.^96^

The *in vitro* and *in vivo* nanotoxicity of metallic NPs is influenced by a host of factors such as their sizes, shapes, redox properties, surface chemistry, chemical stability or propensity to dissolve under certain environmental conditions.^92,93,99^ The latter point is key: the toxicity of a pristine metallic NP will not be the same as a metallic NP that interacts with and is influenced by its environment.^100^ As shown in Scheme 5, the chemisorption of nucleophiles or electrophiles in solution, such as inorganic ligands, can dramatically increase the likelihood of a metallic NP dissolving to release ions. A body of research is emerging relating the toxicity mechanisms for different metallic NPs with the release of toxic ions.^101^ Indeed, metallic NPs that were unable to release toxic ions (for example by surrounding them in a stable coating) were much less toxic.^101^ Even if a metallic NP is inert in solution (so it does not dissolve) its Fermi level in solution may shift considerably after equilibration with redox species. This means that the redox properties at the surface of the metallic NP may change from benign to toxic by, for example, facilitating the production of reactive oxygen species and dramatically increasing the amounts of free-radicals produced when the metallic NP is charged. Similarly, as the size of a metallic NP decreases, its Fermi level and associated redox properties vary. Thus, unsurprisingly, in certain instances smaller NPs were generally shown to be more toxic than larger metallic NPs.^99^ Accordingly, the theoretical framework introduced in this perspective to comprehend the charging and discharging of metallic NPs will be of considerable use to explain the nanotoxicity mechanisms of metallic NPs.

**Fig. 7:**
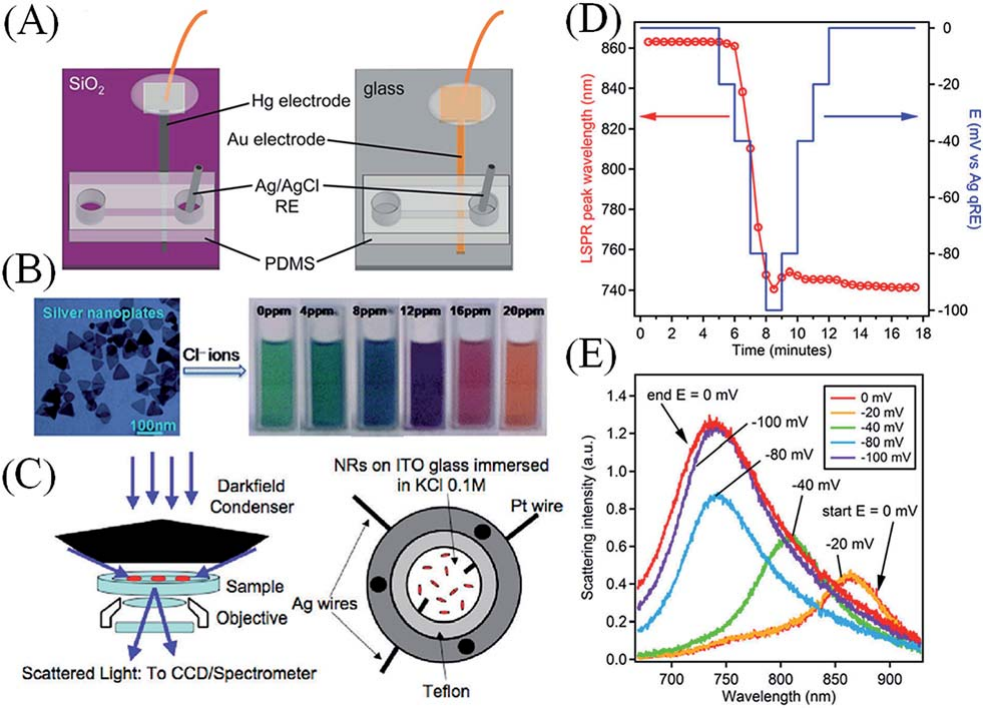
(A) Lab-on-a-chip devices for the analytical detection of metallic NPs: microfluidic devices with either Hg (on the left) or Au (on the right) electrodes for the detection of metallic NP impact events under flowing conditions in the presence of a redox species in solution by electrocatalytic amplification. Adapted with permission from ref. 97; T. M. Alligrant, M. J. Anderson, R. Dasari, K. J. Stevenson and R. M. Crooks, *Langmuir*, 2014, **30**, 13462–13469. Copyright 2014 American Chemical Society. (B) Electrochromic sensors: Ag nanoplates may be used in colorometric sensing applications by monitoring spectral shifts in their plasmon absorbance due to Fermi level equilibration with chemisorbed inorganic anions. Adapted with permission from ref. 87; X. C. Jiang and A. B. Yu, *Langmuir*, 2008, **24**, 4300–4309. Copyright 2008 American Chemical Society. (C) Surface plasmon spectroscopy (SPS) of single metallic NPs: (top left) dark-field microscopy experimental setup consisting of a dark-field microscope with dark-field condenser, objective, CCD camera, and spectrometer and (top right) a cell for electrochemical charging under the dark-field microscope, comprising a steel shell with two Ag wires (one contacting the ITO to provide a working electrode, the other a quasi-reference) and an auxiliary Pt electrode. Adapted with permission from ref. 16; C. Novo, A. M. Funston, A. K. Gooding and P. Mulvaney, *J. Am. Chem. Soc.*, 2009, **131**, 14664–14666. Copyright 2009 American Chemical Society. Monitoring electrodeposition on single metallic NPs: (D) the position of the surface plasmon band peak of a single Au nanostar as a function of both time (red circles) and applied potential (blue line). The measurements were taken during electrodeposition of metallic Ag from an aqueous solution containing 6.7 × 10^–7^ M AgNO_3_ and 0.1 M NaNO_3_. (E) Selected Rayleigh scattering spectra of the same gold nanostar collected at various applied potentials during the deposition process. The nanostar was coated with polyvinylpyrrolidone (PVP). (D and E) adapted with permission from ref. 98; M. Chirea, S. S. E. Collins, X. Wei and P. Mulvaney, *J. Phys. Chem. Lett.*, 2014, **5**, 4331–4335. Copyright 2014 American Chemical Society.

Particularly promising avenues of research allowing the analytical determination of metallic NPs are NP landing or impact studies, either at polarised UME surfaces or using SECCM. These techniques, entirely based on Fermi-level equilibration of NPs with polarised electrode surfaces and redox species in solution, are expected to lead to the development of highly automated, cost-effective, and rapid screening micro-electrochemical devices for much needed point-of-care environmental monitoring systems. A key step in this process will be the development of lab-on-a-chip microfluidic devices capable of quantifying NP impacts. Recent steps in this direction have been made by the groups of Pumera and Crooks. Pumera and co-workers detected Ag NPs in a microfluidic lab-on-a-chip device by electrochemically oxidising (*i.e.*, dissolving) the Ag NPs on impact with an embedded electrode.^96^ Ag NPs of 10 and 40 nm in size were detected, although not as individual NPs but as groups of NPs undergoing simultaneous oxidation. One potential perspective of this work is to multiplex it with micellar electrokinetic chromatography to allow the separation and detection of various sizes of Ag NPs.^102^ Crooks and co-workers developed two microfluidic devices with either Hg or Au electrodes, see Fig. 7A, to carry out electrocatalytic amplification studies under flowing conditions monitoring the collision dynamics of Pt NPs with N_2_H_4_ as the sacrificial redox molecule.^97^ These devices demonstrated several advantages over conventional electrochemical cells including lower limits of detection, higher collision frequencies and more stable electrochemical responses (flat baselines and uniform current transients) over long periods of time.^97^ A possible perspective suggested by Crooks and co-workers is to further enhance the principle advantage of these devices, the high collision frequencies observed, by using NPs with magnetic core/catalytic shell structures and applying magnetic fields to enhance mass transfer.^103,104^ This field is as yet in its infancy with fundamental issues, such as a reported irregular distribution of metallic NPs within the flow-profile,^97^ to be overcome but holds huge promise, in particular if multiplexed with efficient metallic NP separation techniques.

The power of SECCM, as developed by Unwin's group, lies firstly in its ability to determine the catalytic activity of a single NP within an ensemble of NPs.^105^ Within an ensemble of NPs large variations in morphology and catalytic activity are possible, thus obscuring the discrimination of the truly catalytic NPs from those less so. The nanoscale resolution of SECCM, in conjunction with its ease of multiplexing with TEM for example, will give unprecedented access to the structure–activity relationship of a single NP without interference from the “ensemble”. The second advantage of SECCM lies in its flexibility regarding the choice of substrate that the metallic NPs impact onto due to its inherently different mode of operation compared to the UME approach. SECCM forms a nanoscale electrochemical cell by contacting a nanodroplet at the tip of a nanopipette with a macroelectrode.^106^ This means that it is not constrained by the need to fabricate an UME. Thus, interesting substrates that lack an amenable technique to form an UME, such as transition metal dichalcogenides,^107^ may now be assessed by SECCM. This will lead to a whole host of studies to determine the influence of the underlying “inert” substrate on the catalytic activity of either an impacting or electrodeposited NP. Such effects are real as evident in the difference in electrocatalytic activity of Au NPs supported on carbon and on titania in the CO oxidation reaction.^108^ These fundamental studies are key to the development of a complete understanding of advanced nanocomposite materials that may potentially impact the field of electrocatalysis, and in turn fuel cell and solar cell technologies, amongst others.

As noted earlier, surface plasmon spectroscopy (SPS) can monitor changes in the Fermi levels of metallic NPs that exhibit well-defined surface plasmon modes. This principle may be utilized to develop a new class of colorimetric sensors for the sensing of species of interest in solution. For example, in a study by Jiang and Yu,^87^ inorganic anions were detected in the presence of Ag nanoplates by monitoring the shifts in the SPR absorption peak (and therefore colour) after Fermi level equilibration between the Ag nanoplates and chemisorbed inorganic anions (see Fig. 7B). Although, in that study individual inorganic anions could be distinguished from others in a mixture or from inorganic cations, much future work is needed to improve the selectivity of such sensors.

The current state-of-the-art in SPS seeks to use the technique to study electrochemical processes, such as redox reactions or electrodeposition, at single metallic NPs. Mulvaney and co-workers have used dark-field microscopy (DFM) to study the scattered light from single Au nanorods (see Fig. 7C for details of the experimental setup).^16^ DFM permitted the modulation of the optical properties of single Au nanorods to be observed after electrochemical charge injection *via* an ITO electrode.^16^ Using this approach, the kinetics of the charging and discharging of single Au nanorods were directly observed during a redox reaction involving the oxidation of ascorbic acid on the surface of the Au nanorods.^91^ These results constituted the first direct measurement of the rates of redox catalysis on single metallic nanocrystals.^91^ Recently, Mulvaney and co-workers monitored the electrodeposition of metallic Ag onto Au nanostars absorbed to ITO electrodes by DFM and SEM and accurately modelled their observations with COMSOL simulations (Fig. 7D and E).^98^

Clearly, as discussed regarding SECCM, the transition from studying ensembles of NPs to single NPs will allow the effects of NP size, shape and interaction with the substrate on the rates of catalysis to be precisely determined instead of being obscured by incorporation into ensemble averages. This makes SPS a powerful new tool for researchers at the forefront of electrocatalyst development. For further information on the optical characterisation of single plasmonic NPs using alternative techniques to SPS, the reader is referred to recent reports from the groups of Link^109^ and Tao,^110^ and in particular to an informative tutorial review on the topic.^111^

As SPS is a non-invasive optical technique that is influenced by the redox environment within which the metallic NP finds itself, much future work is envisioned where SPS will indirectly relate the redox conditions inside biological tissues and cells optically in a reversible manner over a wide potential range. Regulation of the intracellular redox potential is critically important to cell function.^112^ The disruption of intracellular redox potential may be implicated in the initiation and progression of several disease states including cancer, cardiovascular, neurodegenerative, and autoimmune diseases.^112^ However, despite the importance of the intracellular redox potential as a potential diagnostic marker, its study is severely limited by the lack of suitable measurement techniques. A current state-of-the-art technique involves using an optical approach to infer the intracellular redox potential,^113^ hypothetically demonstrating the viability of SPS in a cellular environment. Briefly, Au nanoshells modified with molecules whose surface enhanced Raman spectroscopy (SERS) spectrum change depending on oxidation state may be controllably delivered to the cytoplasm without any toxic effects.^113^ Then, by accurately measuring the proportions of oxidized and reduced species optically with SERS, the intracellular redox potential can be calculated using the Nernst equation.^113^ Similarly, the shifts in SPR of metallic NPs or nanorods or nanostars or nanoplates *etc.* may be potentially monitored optically in real-time, perhaps by DFM as discussed above, allowing measurement of the changes in the redox environment in the cell.

SPS studies also represent the first steps in the development of a new class of “plasmonic electrochromic” devices, *i.e.*, capable of dynamic colour changes by electrochemical manipulation of the plasmon absorbance of metallic NPs under an applied potential.^84,114^ Interest in the development of plasmonic electrochromic devices has surged in recent years by replacing metallic NPs with transparent conductive oxide (TCO) nanocrystals.^84,114^ TCO nanocrystals may be charged and discharged by Fermi level equilibration with an electrode, such as transparent ITO. However, TCO nanocrystals have much lower carrier concentrations than metallic NPs and, as a result, for the same value of Δ*n* dramatic increases in Δ*λ* are observed. Thus, once more, a field whose history is steeped in the fundamental background of understanding the nanoscale charging and discharging of metallic NPs has taken an unexpected and highly productive turn by applying that theory to new non-metallic nanomaterials. These TCO nanocrystal-based plasmonic electrochromic devices hold huge promise for the development of highly advanced electrochromic windows capable of an independent regulation of transmitted visible light and solar heat into a building by controllably shifting the plasmon absorption from the visible to the near-infrared (NIR) regions of the solar spectrum under an applied potential.^84,114^ A major perspective in this field is the development of new TCO nanocrystals with plasmon absorption peak wavelengths closer to 1250 nm and their integration into devices of low manufacturing cost, long cycle life and capable of both high optical contrast and fast switching times.

The recently demonstrated technique to form gold metal liquid-like droplets by Fermi level equilibration and subsequent adsorption of tetrathiafulvalene with citrate stabilized Au NPs^53^ is expected to lead to applications in optics (as filters and mirrors),^115,116^ biomedical research (size-selective membranes for dialysis, or drug-delivery capsules), model systems to probe the collapse and folding of biological membranes and cellular structures, sensors (SERS at fluid interfaces), catalysis (nanoscale bioreactors) and perhaps as an alternative gold recovery method in the mining industry. The Fermi level equilibration will provide another approach to rationalize the observed phenomena, and help to further develop these systems.

## Conclusions

All in all, it is clear that a good understanding of the redox properties of metallic NPs is essential to develop new processes based on charge transfer reactions. In this perspective we have shown that the behaviour of metallic NPs can be explained by simple thermodynamic and electrostatic models. Fermi level equilibration of NPs with their surroundings will always take place, and will in turn significantly impact the NPs reactivity and optical properties. These effects can be beneficial (for example SERS and SPS sensors) or detrimental (effects on nanotoxicity), and thus must always be taken into account. In the case of metallic NPs, it is important to keep in mind that the charge of the NPs is a very critical parameter, having significant influence not only on the chemical and electrochemical properties of the NPs, but also their physical and biological properties.
